# Associations between Cues of Sexual Desire and Sexual Attitudes in Portuguese Women

**DOI:** 10.3390/ejihpe11040094

**Published:** 2021-10-19

**Authors:** Juliana Silva, Susana Ferreira, Vanessa Barros, Ana Mourão, Gabriela Corrêa, Sónia Caridade, Hélder Fernando Pedrosa e Sousa, Maria Alzira Pimenta Dinis, Ângela Leite

**Affiliations:** 1 School of Human and Social Sciences, Department of Education and Psychology, University of Trás-os-Montes and Alto Douro (UTAD), Quinta de Prados, 5001-801 Vila Real, Portugal; al68328@utad.eu (J.S.); csferreira@chtmad.min-saude.pt (S.F.); al67468@utad.eu (V.B.); al68167@utad.eu (A.M.); al70512@alunos.utad.pt (G.C.); angelal@utad.pt (Â.L.); 2 School of Psychology, University of Minho, Campus de Gualtar, 4710-047 Braga, Portugal; 3 Interdisciplinary Center for Gender Studies (CIEG) of the Higher Institute of Social and Political Sciences of the University of Lisbon (ISCSP-UL), 1300-663 Lisboa, Portugal; 4 Psychology Research Center, School of Psychology, University of Minho, 4710-057 Braga, Portugal; 5 Department of Mathematics (DM.UTAD), University of Trás-os-Montes and Alto Douro (UTAD), Quinta de Prados, 5001-801 Vila Real, Portugal; hfps@utad.pt; 6 UFP Energy, Environment and Health Research Unit (FP-ENAS), University Fernando Pessoa (UFP), Praça 9 de Abril 349, 4249-004 Porto, Portugal

**Keywords:** sexual attitudes, cues of sexual desire, women, brief sexual attitude scale (BSAS), cues of sexual desire scale (CSDS)

## Abstract

Sexuality is defined as a multidimensional experience that involves genital, mental, and bodily components. It is also assumed as a basic condition inherent to the human existence that encourages the search for love, intimacy, sex, and proximity to others. The main objective of this study is to assess the relationship between cues of sexual desire and sexual attitudes in Portuguese women. This is a cross-sectional study with 804 Portuguese women to whom the protocol was applied. It included an informed consent, a sociodemographic questionnaire, a questionnaire related to intimacy, a scale of sexual attitudes, and the scale of cues of sexual desire. The protocol was applied via Google Forms due to the current pandemic situation (COVID-19). Differences were found in sexual attitudes and the cues of sexual desire in terms of sociodemographic characteristics, as well as in terms of women’s intimacy. Significant correlations were found between the brief sexual attitudes scale (BSAS) and the cues of sexual desire scale (CSDS). Age, sexual orientation, relation nature, sexual practices, visual proximity cues, erotic explicit cues, and sensory explicit cues explain, altogether, 25% of the total sexual attitudes. Additionally, age, sexual orientation, the relation’s nature, sexual practices, visual proximity cues, emotional bonding cues, romantic implicit cues, erotic explicit cues, and sensory explicit cues explain, altogether, 30% of the permissiveness. Sexual attitudes are developed under the influence of sociodemographic variables, variables related to women’s intimacy, and cues of sexual desire, which are new data in the study of sexual attitudes and have implications at the level of gender issues.

## 1. Introduction

Sexuality is a central organizing construct that includes attitudes, behaviours, and values aligned with one’s sex, gender, and sexual identity, eroticism, pleasure, intimacy, reproduction, desire, and the like [1]. Sexuality is determined by psychological influences, cultural and social influences, and biological and evolutionary influences [2]. Sexuality influences physical and mental health, being an important predictor of quality of life and corresponding to a globalizing experience in which, in addition to the genital component, the mental and bodily components are also included [3]. Sexuality is shaped by individual values, attitudes, behaviour, physical appearance, emotions, personality, empathy, aversions, spiritual beliefs, and the different influences of the social environment [4,5]. Gray and Garcia [6] studied sexual behaviours through an evolutionary perspective and reported the relative costs and benefits concerning a particular aspect of an organism’s sexual behaviour, relating sexual behaviours to Hamilton’s rule [7], i.e., to be favoured by natural and sexual selection, a behaviour or cognitive process must provide more benefits than costs to the organism in the domains of survival and reproduction.

The World Health Organization [8] defines gender as the characteristics of women and men that are socially constructed (sex refers to those that are biologically determined). “People are born female or male but learn to be girls and boys who grow into women and men. This learned behaviour makes up gender identity and determines gender roles [7]. In 1990, Judith Butler stated that society created a “gender trouble”, assuming that gender is not an essential, biologically determined quality or an inherent identity. “The prevalent view is that biological sex is binary (male vs. female), essential, and natural, and that it forms the basis for binary gender, which is viewed as the cultural interpretation of sex, and sexual desire” [9]. The “gender binary” framework, that assumes that humans comprise two types of beings, women and men, has been strongly challenged by academic research and activism [10]. Findings from neurosciences, behaviour neuroendocrinology, psychology, and development undermine the gender binary [10]. The same authors suggested that “the gender binary should be replaced by a conception of gender/sex that stresses multiplicity and diversity, including a multiple-category (…) system, whose categories are not mutually exclusive (…), fluid (…), and allow for the possibility that gender is viewed as irrelevant to the self”. At the social level, sexuality is experienced in a context in which women struggle for empowerment and equal rights [11], thus emerging a new perspective on the purpose of sex, not being only reproductive but also hedonic [12]. There is a general tendency to consider the female biological sexual response to be universal and almost intuitive. 

Sexual attitudes refer to the attitudes one has toward sexuality or sexual behaviours [13,14]. These attitudes, manifested in a person’s individual sexual behaviour, are influenced by family and cultural perspectives about sexuality, by sexual education, and by prior sexual experiences [15]. These attitudes are also related to sociodemographic characteristics, mainly, race/ethnicity, education, and gender [16,17]. Sexual attitudes play a fundamental role in understanding sexual behaviour (sexual desire and satisfaction), as the attitudes themselves shape cognitions, intentions, and behaviours [18]. Thus, a positive sexual attitude can be responsible for the positive assessment of the sexual experience itself and for the presence of several cues of sexual desire. Sexual attitudes predict partnered sex and sexual interest, that is, if someone thinks about sex often and rates sex as important, they naturally also have sex more often [15]. Women’s sexual attitudes may influence their judgments, namely, of victimization risk, i.e., women who report positive attitudes about casual and impersonal sex tend to consider hypothetical dating situations as less risky than women who report fewer positive attitudes, i.e., women with more positive attitudes toward casual sex are at an increased risk of sexual victimization [19]. Women tend to more easily refuse sex without commitment, while men, due to the positive expectations generally associated with this type of behaviour, tend more often to these sexual practices [20,21]. According to Antunes [22], women do not seek the satisfaction of physical pleasure as much as the satisfaction of emotional needs. In addition, the same author found that people who are not in a relationship tend to be more open and permissive than those who are in a relationship, which tend to be more affectionate and idealistic. Attitudes concerning human sexual expression are described as liberal or conservative orientation in a single bipolar continuum [23]. More liberal sexual attitudes and the willing to experience romantic/passionate emotions were associated with higher levels of desire [24]. Concerning masturbation, pornography, premarital, and extra-relationship sex, women are more sexually conservative than men, but more tolerant towards homosexuality, and more approving of abortion [25].

Sexual desire motivates individuals to engage in sexual activity [26] and it is triggered by physiological, neurological, and hormonal changes that accompany sexual arousal [27]. Sexual activity is associated with benefits and costs [28]. Accordingly, benefits include pleasure, stress reduction, goal attainment, and emotional commitment, and costs include transmission of sexually transmitted infections, unwanted pregnancies, and reputational damage.

As natural selection requires variation in psychological traits, women naturally differ relating this specific aspect [28]. Concerning higher or lower levels of sexual desire, natural selection should select for a moderate level of sexual desire as both extremes have costs. Being sexual desire context-dependent, it should vary in response to adaptive problems, e.g., procuring a mate, parenting, and mate retention, and contextual factors, e.g., having children [28,29]. It is challenging to comprehend individual variation in sexual desire in response to McCall and Meston [30] term cues for sexual desire, namely, emotional bonding cues, e.g., feeling a sense of love or commitment; visual/proximity cues, e.g., someone acting confidently; explicit/erotic cues, e.g., talking about sex or having a sexual fantasy; and implicit/romantic cues, e.g., having a romantic dinner with a partner. McCall and Meston [30] also found that women with low sexual desire reported fewer love/emotional bonding cues, erotic/explicit cues, and romantic/implicit cues, when compared with women with no sexual concerns. The same authors also found that postmenopausal women endorsed more love/emotional bonding cues, which resulted in sexual desire, when compared with premenopausal women. Barreto et al. [31] found that sexual cues significantly predict sexual desire, in particular, explicit/arousal cues were the most significant predictor of female sexual desire. The influence of attitudes in triggering desire is done in two ways: implicitly or automatically, and explicitly or in a controlled way. For example, a woman may feel sexual desire due to a certain cue, but this desire can be controlled as a function of other stimuli [18]. The individual factors are another relevant aspect. Once effective cues of sexual desire in the past, may not prove to be functional at present. Likewise, cues of sexual desire with a strong effect on one partner may not be effective with another sexual partner [32].

With regard to the relationship between sex and commitment, there is the so-called “casual sex”, whose main characteristic is the absence of a relational commitment between people or just the existence of a friendship. In this regard, Alvaréz et al. [33] concluded that relationships without commitment are solely for sexual purposes, implying that they tend to have higher rates of cues of sexual desire. Jonason et al. [34] report that factors such as insecure attachment, psychopathy, and narcissism are positively associated with casual sexual relationships. Regarding committed relationships, the same authors also claim that these offer emotional support and that individuals with safer attitudes towards love and sex tend to despise the practice of casual sex.

According to Rosemary Basson’s Non-Linear Model of Sexual Response [35], most women start from a process of sexual neutrality and, when emotional intimacy and/or stimulation arises, a sexual cycle is generated that includes among other components, satisfaction, desire, and excitement, The desire may initially be absent, but it may be triggered by the process of sexual arousal. Some parts of the model are linear, e.g., arousal and stimulation occur prior to the experience of satisfaction, and other parts are circular and bidirectional, e.g., sexual desire may come before or after arousal feeding into each other. When exposed to cues relevant to sex, e.g., kissing or petting, women may feel sexually aroused, which might then lead to sexual desire [35]. How long a relationship lasts also influences the frequency of sexual activity. Women in long-term relationships, i.e., more than five years, have fewer cues of sexual desire, when compared to women in shorter relationship, i.e., less than three years [32]. It is then necessary to understand how women interpret and perceive cues to consent to sexual behaviours in a given situation and how these are influenced by the duration of the relationship. Since 1999 and 2011 the Sexual Attitudes Scale (SAS) [36] and Cues for Sexual Desire Scale (CSDS) [30] have not been applied to samples of Portuguese women. A long time has passed since then and social, cultural, and psychological changes have certainly impacted attitudes towards sexuality. There is a coexistence of highly gendered sexual scripts with increasingly egalitarian sexual roles, namely among the youngest and the most educated generations in Portuguese society [37]. However, data on Portuguese young women show that dysfunctional sexual attitudes, such as conservative and negative attitudes towards sexuality persist and interfere with sexual functioning [38]. Traeen et al. [39] found that 63% of women rated sex as moderately or very important. Portuguese women believe sex is good for well-being and do not believe ageing is an obstacle to sexual enjoyment in older adults [40]. Thus, the main objective of this study is to assess the relationship between the cues of sexual desire and sexual attitudes in Portuguese women. To achieve this goal, the sample is characterized, and the questionnaire related to women’s intimacy is analysed. The psychometric characteristics of the instruments used are identified. The means of the dimensions studied are compared as a function of sociodemographic variables and the variables of the questionnaire on women’s intimacy. Correlations are established between the two constructs and, finally, the variables that best explain sexual attitudes in Portuguese women are identified.

Considering that the assumptions of good psychometric qualities of the instruments are guaranteed (described below), the following hypotheses were established:

**Hypothesis** **1** **(H1).** *It is expected that the cues of sexual desire and sexual attitudes vary according to age, marital status, and educational qualifications, as well as according to sexual orientation, the duration of the relationship, and the nature of the relationship.*

**Hypothesis** **2** **(H2).** *It is expected to find significant and positive correlations between cues of sexual desire scale (CSDS) and sexual attitudes (BSAS) and respective subscales.*

**Hypothesis** **3** **(H3).** *It is expected that the sexual attitudes of Portuguese women are explained in a multifactorial way: by sociodemographic characteristics, by characteristics of intimacy, and by the cues of sexual desire scale (CSDS).*

## 2. Materials and Methods

This study has a descriptive, correlational character, since it intends to describe the relationships found between variables. It is also transversal, i.e., single application, and quantitative (statistical treatment of the collected data is carried out) in nature. 

### 2.1. Sample

Based on the analysis of questionnaires obtained from 851 individuals aged between 17 and 70 years old, and after applying the inclusion criteria, i.e., being female, being aged between 18 and 74 years old, and having Portuguese nationality, the sample of this study is composed by 804 Portuguese women with a mean age of approximately 26 years (26.05 ± 10.19 (*min* = 18; *max* = 71)). For the purposes of statistical analysis, age was converted into a variable divided into four groups: 1–18 to 20 years, *n* = 102 (12.7%); 2–21 to 30 years, *n* = 325 (40.4%); 3–31 to 40 years, *n* = 197 (24.5%); 4–41 to 71 years, *n* = 180 (22.4%).

Most of the sample is not in a relationship (single: *n* = 612 (76.1%), is married/in an unmarried union: *n* = 158 (19.7%), and divorced: *n* = 34 (4.2%)). Concerning education, the majority of the sample is concentrated in higher education, *n* = 464 (57.7%). With regard to their professional status, 481 women (59.8%) are students and the remaining work.

Regarding sexual orientation, most of the sample is heterosexual, 702 women (87.3%). Participants are at the two extremes in terms of relational duration, i.e., 322 (40%) have been in a relationship for more than 24 months and 282 (35.1%) are not in a relationship, while 59 (7.3%) women are in a relationship for about six months, 51 (6.3%) for 6 to 12 months, and 90 (11.2%) for 12 to 24 months.

Considering the relational nature, the following data were obtained: 483 (60.1%) women are in a committed relationship, 282 (35.1%) are not in a relationship, and 39 (4.9%) are in a non-committed relationship. In regard to the practice of sexual relations in the last month, there were 516 (64.2%) negative responses and 288 (35.8%) positive ones. Finally, 533 women (66.3%) stated that they only maintain current sexual practices with a single partner, in contrast to 16 women (2%) who admit practices with multiple sexual partners. In addition, 115 women (14.3%) report current sexual practices without a partner, e.g., masturbation, and 140 (17.4%) report not having any type of sexual activity.

### 2.2. Procedure

Considering the most up-to-date version of the Declaration of Helsinki [41] and all the ethical principles underlying it, a research protocol was created, including sociodemographic questionnaire and a questionnaire related to women’s intimate lives, CSDS, and BSAS, which was only applied after the participants signed the informed consent including the purpose of the study. It functions as a contractual document where all ethical principles of research in social and human sciences are ensured, guaranteeing that all information collected is only used to pursue the research objectives. In the introduction of the protocol, it is explained that it is intended for women, over 18 years of age, and with Portuguese nationality. To that extent, the authors assume that respondents considered themselves, or felt themselves to be, women.

The protocol was made available online through Google Forms due to the current pandemic period (COVID-19) and was posted on a page on a social network created specifically for this purpose. Its dissemination was made mainly through personal contacts, using social networks and other digital means, obtaining a sample of snowball effect, non-probabilistic for convenience. The collection of the data was carried out during the month of November 2020. The data obtained were processed using the Statistical Package for the Social Sciences (SPSS 27).

### 2.3. Instruments

**Sociodemographic questionnaire and questionnaire on women’s intimacy**. A sociodemographic questionnaire was constructed, including sociodemographic issues (age, marital status, education, and profession); the questionnaire on women’s intimacy includes issues related to the intimacy of women (sexual orientation, duration of the current relationship, nature of the relationship, sexual relations in the last month, current sexual practices, and sexual partners). Regarding the latter, the sexual orientation variable was open and was later recoded as 1—heterosexual and 2—other orientations, given the scarcity of answers for this option. The variable duration of the current relationship included the following answer options as: 1—I am not in a relationship; 2—less than 6 months; 3—6 to 12 months; 4–12 to 24 months; 5—more than 24 months. The relational nature variable allows the following response options: 1—I am not in a relationship; 2—no commitment; 3—with commitment. The variable practice of sexual intercourse in the last month allows answering yes or no. The variable current sexual practices and sexual partners allows choosing one of the following response modalities: 1—no; 2—yes, no sexual partner; 3—yes, with only one sexual partner; 4—yes, with more than one sexual partner.

**Sexual Attitudes Scale (SAS).** The SAS was developed by Hendrick and Hendrick [36], having been validated for the Portuguese population by Alferes [41] and later revised by Antunes [22]. In the present study, the Portuguese version is used in its reduced version (Brief Sexual Attitudes Scale, BSAS), shown to be sufficient and appropriate in relation to the outlined objectives. This scale was developed in favour of the study of sexual patterns in American society in the 1980s and its authors viewed sexual attitudes as a multidimensional concept, therefore seeking to build an instrument that would investigate the complexity of the relationships between sexuality and love [41].

In its original version, SAS presents 43 items organized into four subscales: Permissiveness sexual permissiveness (PER-items 1 to 21), sexual practices (PRA-items 22 to 28), communion (COM-items 29 a 37), and instrumentality (INS-items 38 and 43). In its short version, the BSAS is composed of 22 items and the response modality varies on an ordinal Likert-type scale of five points, scored from 1—“completely in disagreement” to 5—“completely in agreement”, with high values translating positive sexual attitudes [36]. The BSAS short form is divided into four subscales that correspond to the PER, referring to attitudes towards “occasional sex”, “non-committal sex”, and “sexual partner diversity”—items 1, 3, 4, 6, 8, 11, 12, 15, and 20, to COM, addressing attitudes towards sex as a physical, psychological, sharing, emotional involvement, and idealism experience—items 9, 13, 16, 18, and 21, to INS, depicting the attitude towards the idea of sex as a utility, a way of obtaining purely physical pleasure—items 5, 10, 14, 19, and 22, and to PRA, concerning attitudes towards family planning, sex education, and acceptance of practices such as masturbation (unconventional sex)—items 2, 7, and 17. 

BSAS revealed good reliability and validity through Cronbach’s alpha (*α*), i.e., *α* = 0.93 (PER), *α* = 0.71 (COM), *α* = 0.77 (INS), and finally *α* = 0.84 (PRA). In assessment for the Portuguese population, it also demonstrated good internal consistency, i.e., *α* = 0.75 (PER), *α* = 0.70 (COM and PRA) and *α* = 0.64 (INS) [41].

**Cues for Sexual Desire Scale (CSDS).** The initial version of the CSDS [30] includes 125 items resulting from applying a single question to a sample of 50 women aged 18–67 years. The starting question was: “What makes you feel desire for sexual activity?”, with sexual activity being defined as “kissing, caressing, oral sex, sexual intercourse and/or masturbation”. Starting from these 125 items, and using as an exclusion criterion absolute factor values lower than 0.40, a new, reduced version was obtained.

CSDS, proposed by McCall and Meston [30], is composed of 40 items, evenly distributed by four factors (ten items each): the first factor called emotional bonding cues obtained an *α* = 0.92, the second factor designated explicit/erotic cues, with an *α* = 0.87, the third factor called visual/proximity cues, with *α* = 0.87, and finally the fourth factor, the romantic/implicit cues with *α* = 0.88. When applying the scale, the respondent is asked to indicate how likely a certain situation, described in a certain item, arouses sexual desire. The response modality is available on a five-point Likert-type auto-response scale, where “1” corresponds to “unlikely” and “5” to “extremely likely”. A high score on this scale indicates the presence of higher rates of cues of sexual desire.

In the Portuguese version of the CSDS [32], 38 items are presented distributed by five factors (total *α* = 0.91), reformulated as follows: factor one includes eight items (*α* = 0.91), factors two and three contain ten items each (*α* = 0.89 and *α* = 0.88, respectively), and finally, factors four and five contain five items each (*α* = 0.85 and *α* = 0.86, respectively). Compared to the original version, the nomenclature of the factors was revised, with factor three in the original version corresponding to the first factor “visual/proximity clues” in the Portuguese version. The second factor was called “Emotional Bonding Cues”, the third factor “romantic/implicit cues”, and the fourth factor “explicit/erotic cues” from the original scale, splits and corresponds to the fourth and fifth factors, i.e., to “explicit/excitement cues” and “explicit/sensory cues”, respectively. It should be noted that in the Portuguese version, both the response modality and the application instructions remain the same as in the original version.

### 2.4. Data Analysis

Data were processed using the SPSS statistical analysis program, version 27. A descriptive statistical analysis was carried out (minimum, maximum, mean, standard deviation, kurtosis, and skewness), whose indicators allowed to describe the sample and the variables related to the intimacy of women, as well as analysing the instruments used (BSAS and CSDS). Cronbach’s alpha (α) and Split-half were also used to assess the reliability and consistency of the instruments used. An inferential statistical analysis was also carried out, allowing to establish relationships between the different variables, such as correlations (Pearson’s *r*), difference tests (ANOVA for variables with three or more response options; Student’s *t*-test for dichotomous variables), and regressions (multiple hierarchical). Analysis of variance (ANOVA) splits an observed aggregate variability found inside a data set into two parts: systematic factors and random factors. ANOVA test allows a comparison of more than two groups at the same time to determine whether a relationship exists between them [42]. A student’s *t*-test is used as a hypothesis testing tool, which allows testing of an assumption applicable to a population [43]. Hierarchical regression is a form to assess if variables of interest explain a statistically significant amount of variance in the dependent variable after accounting for all other variables. In hierarchical multiple regression analysis, the researcher determines the order in which variables are entered into the regression equation [44].

## 3. Results

### 3.1. Brief Sexual Attitudes Scale and Cues for Sexual Desire Scale

Regarding the BSAS [41], the descriptive statistics of the items, using the normative values proposed by Kline [45], skewness |*sk*| < 3 and kurtosis |*ku*| < 10), allows us to verify that the items in this instrument follow a normal distribution. The descriptive statistical analysis of the CSDS items [32] reveals that they also have a normal distribution. The same happens with the total scale and its dimensions (Table 1).

Table 1 also shows the total mean values of the BSAS and CSDS and their subscales, together with the Cronbach’s alpha and Split-half values; Cronbach’s alpha values were, respectively, higher and identical to the values obtained in the measurement of the Portuguese population. Regarding the BSAS, the sexual practices subscale has the highest average and the instrumentally subscale the lowest. For CSDS, the emotional bonding cues subscale has the highest average and the visual proximity cues subscale the lowest.

### 3.2. Associations and Differences between Brief Sexual Attitudes Scale and Cues for Sexual Desire Scale Means and Their Subscales, According to Sociodemographic Variables

Age is negatively, significantly, and weakly correlated with the total BSAS (*r* = −0.150; *p* < 0.001) and with the permissiveness subscale (*r* = −0.222; *p* < 0.001). These results mean that younger women have higher BSAS and permissiveness values. Statistically significant differences were found in the values of total BSAS as a function of marital status. Single women have higher values than married and divorced women, and these have higher values than married women. It was also found that only in the permissiveness subscale there were statistically significant differences, with higher values in single women (Table 2). Regarding to BSAS, statistically significant differences were found in two subscales, i.e., sexual practices (*t*(804) = −2.431; *p* = 0.015; *d* = −0.174) and communion (*t*(804) = 3.133; *p* = 0.002; *d* = −0.224), depending on educational qualifications. Women with university studies have significantly higher values than those without university studies in both subscales. 

Age is negatively, significantly, and weakly correlated with CSDS total (*r* = −0.165; *p* < 0.001), with visual proximity cues (*r* = −0.107; *p* < 0.010), emotional bonding cues (*r* = −0.147; *p* < 0.001), and erotic explicit cues (*r* = −0.168; *p* < 0.001). These results mean that younger women have higher values in these dimensions. There are statistically significant differences in the values of two CSDS subscales as a function of marital status, i.e., emotional bonding cues (married women have higher values, followed by single women and finally divorced women) and erotic explicit cues (single women have higher values high, followed by married and finally divorced) (Table 2). Furthermore, it is on the permissiveness variable that marital status has a greater impact.

### 3.3. Differences between Brief Sexual Attitudes Scale and Cues for Sexual Desire Scale Means and Their Subscales, According to Variables Related to Women’s Intimacy

#### 3.3.1. Sexual Orientation

Regarding the total BSAS and the permissiveness subscale, it was found that there are statistically significant differences in the values according to sexual orientation, with non-heterosexual women having higher means (Table 3). With regard to total CSDS and all subscales (except emotional bonding cues), it was found that there are statistically significant differences in values depending on sexual orientation, with non-heterosexual women showing higher mean values in all dimensions CSDS (Table 3).

#### 3.3.2. Relationship Time

Regarding relational duration, it was found that there are significant differences in the total BSAS, with women in relationships with less than 6 months showing higher values. It was also found that there are statistically significant differences in relation to permissiveness, with higher values for those who are not in a relationship, and communion, with higher values for women who have been in a relationship for more than 24 months (Table 4). Additionally, in relation to relational duration, statistically significant differences were found in the values of total CSDS, with women in relationships with less than 6 months having higher values. Differences were also found in the emotional bounding cues (the relational duration of 12–24 months has the highest values), erotic explicit cues (the relational duration of 6–12 months has the highest values), visual proximity cues (the relational duration of less than 6 months has the highest values), and romantic implicit cues (the relational duration of 6–12 months has the highest values) (Table 4).

#### 3.3.3. Nature of the Relationship

There are statistically significant differences in the total BSAS and in all subscales except instrumentality, according to the relational nature. Regarding the total BSAS and the permissiveness subscale, it was found that the highest values are registered in the response modality “I am not in a relationship”. However, in the sexual practice and communion subscales the highest values appear in the “without commitment” (Table 5). Additionally, in relation to the total CSDS and all its subscales, with the exception of sensory explicit cues, it was found that there are statistically significant differences in the values depending on the relational nature. On the visual proximity cues subscale, the modality with the highest values is “I am not in a relationship”; in the erotic explicit cues and romantic implicit cues subscales, the modality with the highest values is “I am in a relationship without commitment”. Women who are in a committed relationship score higher on the emotional bonding cues subscale CSDS (Table 5).

#### 3.3.4. Sexual Practices and Current Sexual Partners

Regarding the total BSAS, it was found that there are statistically significant differences in the values in relation to sexual practices and current sexual partners, with women who have more than one sexual partner presenting a higher average. It was also found that there are statistically relevant differences in all subscales, except in sexual practices, highlighting communion in the “Yes, with more than one sexual partner” modality and permissiveness in the “no” modality with higher values (Table 6). Regarding CSDS, statistically significant differences were found according to sexual practices and current sexual partners in all subscales and in total. In total, in the visual proximity cues and in the romantic implicit cues, women with more than one sexual partner score higher in emotional bonding cues, while in the sensory explicit cues and erotic explicit cues subscales women who do not have a sexual partner score higher (Table 6).

### 3.4. Analysis of Correlations between the Cues for Sexual Desire Scale and Brief Sexual Attitudes Scale 

The results showed multiple significant correlations, although mostly moderate and weak. The positive correlations between the CSDS total, sensory explicit cues, erotic explicit cues, visual proximity cues, and the BSAS total are highlighted, as well as the positive correlations between the sensory explicit cues, visual proximity cues, and the BSAS permissiveness subscale (Table 7).

### 3.5. Regression Analyses

Age, sexual orientation, the relation´s nature, sexual practices, visual proximity cues (CSDS), erotic explicit cues (CSDS), and sensory explicit cues (CSDS), altogether, explain 25% of the BSAS (total) variance (*F*[7, 787] = 37.851; *p* < 0.001) (Table 8), being that sensory explicit cues and visual proximity cues are the variables that contribute the most to the BSAS variance.

Age, sexual orientation, the relation´s nature, sexual practices, visual proximity cues (CSDS), emotional bonding cues (CSDS), romantic implicit cues (CSDS), erotic explicit cues (CSDS), and sensory explicit cues (CSDS), altogether, explain 30% of the permissiveness (BSAS) variance (*F*(9, 785) = 39.215; *p* < 0.001) (Table 8), being that the nature of the relation, erotic explicit cues, and sexual practices are the variables that contribute the most to the permissiveness (BSAS) variance.

Age (*β* = −0.149; *t* = −4.742; *p* < 0.001), CSDS (total) (β = 0.114; *t* = 2.905; *p* < 0.01), and visual proximity cues (CSDS) (β = 0.192; *t* = 5.409; *p* < 0.001), altogether, explain 3% (*R*^2^ = 0.029; Δ*R*^2^ = 0.025) of the sexual practices (BSAS) variance (*F*[3, 800] = 8.677; *p* < 0.001), being that visual proximity cues is the variable that contribute the most.

Relation duration (*β* = 0.151; *t* = 2.025; *p* < 0.05), relation nature (*β* = −0.156; *t* = −2.112; *p* < 0.05), visual proximity cues (CSDS) (*β* = 0.266; *t* = 7.498; *p* < 0.001), and sensory explicit cues (CSDS) (*β* = 0.109; *t* = 3.128; *p* < 0.01), altogether, explain 10% (*R*^2^ = 0.101; Δ*R*^2^ = 0.096) of the instrumentality (BSAS) variance (*F*(4, 799) = 22.390; *p* < 0.001), being that visual proximity cues is the variable that contribute the most.

Education (*β* = 0.087; *t* = 6.483; *p* < 0.01), sexual practices (*β* = 0.096; *t* = 2.596; *p* < 0.05), emotional bonding cues (CSDS) (*β* = 0.080; *t* = 2.780; *p* < 0.05), romantic implicit cues (CSDS) (*β* = 0.123; *t* = 2.235; *p* < 0.001), erotic explicit cues (CSDS) (*β* = 0.118; *t* = 3.364; *p* < 0.01), and sensory explicit cues (CSDS) (*β* = 0.102; *t* = 2.727; *p* < 0.05), altogether, explain 11% (*R*^2^ = 0.118; Δ*R*^2^ = 0.112) of the communion (BSAS) variance (*F*(6, 797) = 17.826; *p* < 0.001), being that romantic implicit cues is the variable that contributes the most.

**Figure 1:**
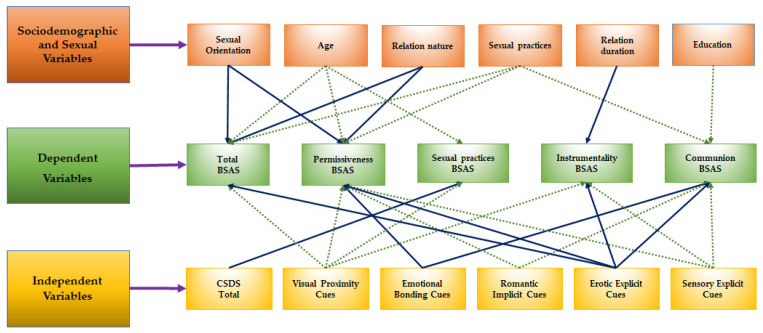
Relationships between sociodemographic and sexual variables, dependent and independent variables. Different arrows are used clarification purposes only.

In Figure 1 it is possible to find the different relationships established between sociodemographic and sexual variables, dependent and independent variables.

## 4. Discussion

The main objective of this study was to assess the relationship between cues of sexual desire and sexual attitudes in Portuguese women.

The instruments used to assess the cues of sexual desire (CSDS) and sexual attitudes (BSAS) in Portuguese women presented good psychometric qualities. In fact, the Cronbach’s alpha values found in this study are very close to the values found in the original versions and in the validation versions for the Portuguese population, and, in some cases, these values are even higher in this study. Cronbach’s alpha (*α*) is used to examine the internal consistency or reliability of summated rating scales [46]. Cronbach’s alpha is used by the authors to demonstrate that tests and scales that have been constructed or adopted for research projects are fit for purpose [47]. Thus, in this study, the reliability of the instruments used was confirmed, considering the objectives of the study.

The first hypothesis (**H1**) predicted that cues of sexual desire and sexual attitudes would vary according to age, marital status, and educational qualifications, as well as according to sexual orientation, the duration of the relationship, and the nature of the relationship. This hypothesis was also confirmed, which is in line with the perspective proposed by Laumann et al. [16] who considered that sexual attitudes were related to sociodemographic characteristics, mainly, race/ethnicity, education, and gender. In this study, younger and single women had higher values in total sexual attitudes and permissiveness, as well as in visual proximity cues, emotional bonding cues, and erotic explicit cues. These results corroborate what was proposed by Pereira et al. [48], who found that older women have more negative and less permissive sexual attitudes than younger women. McCall and Meston [30] also concluded that menopausal and post-menopausal women tend to show a decrease in sexual desire. In addition, females live well beyond their reproductive capacities [49], being clear that, “frequency and diversity of sexual behaviour tend to decline with age, which is consistent with expectations of evolutionary theory and age-related changes in mechanisms of sexual response”.

Women with university studies had significantly higher values than those without university studies in sexual practices and communion, which is in line with Arega et al. [50]. Furthermore, non-heterosexual women have higher means on the BSAS total and permissiveness subscale, as well as on the CSDS and all subscales, with the exception of bonding emotional cues, than heterosexual women, which is in line with Jankowiak and Escasa-Dorne [51], who postulated that bisexual females are more open and expressive than straight females about entering into a casual sex encounter; but contradicts Nimbi et al. [52]. These same authors found that sexual desire levels do not seem significantly different in people who identify as gay or lesbian, when compared to their heterosexual counterparts. Women who are not in a relationship have higher values in permissiveness. Women who have been in relationships for less than six months exhibit higher values of total BSAS, total CSDS, and visual proximity cues. Women who have been in a relationship for between 6 and 12 months score higher on erotic explicit cues and romantic implicit cues. Women who have been in a relationship for between 12 and 24 months have higher values in emotional bounding cues and women who have been in a relationship for more than 24 months score higher in communion. These results corroborate those of Carvalheira et al. [32] who state that women in long-term relationships, i.e., more than five years, have fewer cues of sexual desire when compared to women in shorter relationships, i.e., less than three years. As for the nature of the relationship, women who are in a non-committed relationship score higher on the BSAS sexual practice and communion subscales and on the CSDS erotic explicit cues and romantic implicit cues subscales. Women who are in a committed relationship score higher on the emotional bonding cues subscale.

Women who engaged in sexual practices in the last month scored higher on the communion (BSAS) and erotic explicit cues (CSDS) subscale. However, women who had no recent sex had consistently less well-being, especially for women with more permissive attitudes [53]. Women without a sexual partner score higher on the permissiveness and on the sensory explicit cues and erotic explicit cues subscales. Women who have more than one sexual partner score higher on the total BSAS and its subscales, with the exception of sexual practices, and on the total CSDS, visual proximity cues, and romantic implicit cues. These results corroborate those of Jankowiak et al. [54], who found that “females’ motivation for seeking casual sex ranged from a desire to be validated as being sexually desirable, i.e., rebound sex, seeking a bisexual experience, and having a heighten sex drive”. Women who have a sexual partner score higher on communion and instrumentality and also on emotional bonding cues. 

The second hypothesis (**H2**) predicted finding significant and positive correlations between cues of sexual desire (CDSD) and sexual attitudes (BSAS) and respective subscales, which was confirmed. In fact, the results of this study showed multiple significant correlations, although mostly moderate and weak. The positive correlations between the CSDS total, sensory explicit cues, erotic explicit cues, visual proximity cues, and the BSAS total are highlighted, as well as the positive correlations between the sensory explicit cues, visual proximity cues, and the BSAS permissiveness subscale. This is in line with the literature that suggests that a positive sexual attitude can be responsible for the positive assessment of one’s own sexual experience and for the presence of various cues of sexual desire [18,30,55,56]. In fact, “humans evolved a pluralistic mating repertoire that differs in adaptive ways across sex and temporal context, personal characteristics such as mate value and ovulatory status, and facultative features of culture and local ecology” [57]. Women’s mating strategies tend to change over the cycle, with short-term mating desires and behaviours being stronger in the highly fertile days before ovulation [58].

Finally, the third hypothesis (**H3**) predicted that the sexual attitudes of Portuguese women would be explained in a multifactorial way: by sociodemographic characteristics, by characteristics of intimacy, and by the cues of sexual desire. This hypothesis was also confirmed, especially in relation to the total BSAS and the BSAS permissiveness subscale, as in relation to the other dimensions of the BSAS. The values explained by the regressions were equal to or less than 11%, which is clearly not significant. Thus, age, sexual orientation, the relation’s nature, sexual practices, visual proximity cues (CDSD), erotic explicit cues (CDSD), and sensory explicit cues (CDSD) explain, altogether 25% of the BSAS (total). These results are in line with the literature. In fact, several authors have reported the impact of sociodemographic variables on sexual attitudes [59,60], as well as that of sexual practices on sexual attitudes [61]. However, the type of cues of sexual desire that impacts sexual attitudes is not studied in the literature. In addition, age, sexual orientation, the relation’s nature, sexual practices, visual proximity cues (CDSD), emotional bonding cues (CDSD) (negatively), romantic implicit cues (CDSD) (negatively), erotic explicit cues (CDSD), and sensory explicit cues (CDSD), altogether, explain 30% of the permissiveness (BSAS) variance. Without a doubt, sex encourages and romance discourages sexual permissiveness [62]. 

## 5. Conclusions

Sexual attitudes are developed under the influence of sociodemographic variables, variables related to women’s intimacy and cues of sexual desire, which are new data in the study of sexual attitudes, and has implications at the level of gender issues. However, several limitations were found to the continuation of this investigation, namely the fact that this is a “taboo” topic, especially with regard to females, which made the collection of the sample difficult, despite the confidentiality and anonymity of the data in the informed consent. It should also be noted that the sample was a convenience one, which prevents generalization and the guarantee of representativeness of the sample. Furthermore, the fact that self-report measures were used did not allow for the control of social desirability. Given the current pandemic situation (COVID-19), digital means were used to apply the questionnaire, through Google forms, which in turn made it impossible to clarify doubts. Due to the cross-sectional nature of the study, the relevance of conducting a longitudinal investigation is highlighted, as data collected in more than one moment and over a longer period of time will make it possible to establish stable and reliable relationships between the variables without the interference of biased information and verify possible changes in the participants’ answers. The application of this research to an older female population is also suggested. Finally, a new measurement of both scales aimed at the Portuguese population is recommended, considering the social and behavioural changes that have taken place in recent decades, thus allowing for more up-to-date and truthful results.

## Figures and Tables

**Table 1:** Descriptive statistics of total Brief Sexual Attitudes Scale and Cues for Sexual Desire Scale and respective subscales, Cronbach’s alpha and Split-half of this study, original studies, and validation studies for the Portuguese population (*N* = 804).

				Statistics					
Instruments	*M ± SD*	*Min*	*Max*	*Sk* (0.086)	*Ku* (0.172)	Split- Half	α	α *	α **
**BSAS**									
BSAS total	3.28 ± 0.53	1.45	4.73	0027	−0.580	0.62	0.81	0.71–0.93	0.64–0.75
Permissiveness	2.83 ± 4.53	1.00	4.78	−0.152	−0.069	0.69	0.86	0.93	0.75
Sexual Practices	4.53 ± 0.68	1.00	5.00	−0.129	−0.873	0.62	0.70	0.84	0.70
Instrumentality	2.70 ± 0.79	1.00	5.00	−1.944	4.371	0.54	0.66	0.77	0.64
Communion	3.92 ± 0.69	1.40	5.00	0.130	−0.351	0.41	0.66	0.71	0.70
**CSDS**									
CSDS total	3.48 ± 0.51	1.34	4.95	−0.473	0.860	0.37	0.90	-	0.91
Visual Proximity Cues	2.37 ± 0.89	1.00	5.00	0.353	−0.543	0.79	0.90	0.87–0.88	0.91
Emotional Bonding Cues	4.39 ± 0.63	1.00	5.00	−1.438	2.543	0.77	0.90	0.90–0.93	0.89
Romantic Implicit Cues	3.47 ± 0.81	1.00	5.00	−0.410	0.020	0.71	0.87	0.86–0.89	0.88
Erotic Explicit Cues	4.00 ± 0.83	1.00	5.00	−0.971	0.848	0.68	0.84	0.78–0.90	0.85
Sensory Explicit Cues	2.93 ± 0.93	1.00	5.00	0.027	−0.580	0.56	0.78	-	0.86

Note: *N* = frequencies; *M* = mean; *SD* = standard deviation; *Min* = minimum; *Max* = maximum; *α* = Cronbach’s alpha; α * = values referring to the instrument validation study (Adapted from [30,36]); α ** = values referring to the adaptation study for the Portuguese population [32,41], *Sk* = skewness, *Ku* = kurtosis, BSAS—Brief Sexual Attitudes Scale; CSDS—Cues for Sexual Desire Scale.

**Table 2:** Comparison of Brief Sexual Attitudes Scale and Cues of Sexual Desire Scale means and subscales concerning marital status.

Instruments	Single	Married	Divorced	*F* (804)	*η^2^*
	*M (SD)*	*M (SD)*	*M (SD)*		
**BSAS**					
BSAS total	3.32 (0.52)	3.11 (0.55)	3.23 (0.53)	5.89 ***	0.16
Permissiveness	2.95 (0.89)	2.37 (0.90)	2.74 (0.81)	43.07 ***	0.25
Sexual practices	4.52 (0.71)	4.56 (0.57)	4.53 (0.64)	0.24	0.03
Instrumentality	2.70 (0.80)	2.73 (0.74)	2.61 (0.59)	0.43	0.03
Communion	3.91 (0.67)	3.95 (0.74)	3.95 (0.77)	0.27	0.03
**CSDS**					
CSDS total	3.50(0.50)	3.42 (0.52)	3.37 (0.63)	1.19	0.08
Visual Proximity Cues	2.42 (0.87)	2.18 (0.95)	2.36 (0.90)	7.02	0.11
Emotional Bonding Cues	4.39 (0.61)	4.47 (0.59)	3.98 (0.94)	6.54 ***	0.14
Romantic Implicit Cues	3.48 (0.82)	3.45 (0.80)	3.52 (0.73)	0.16	0.02
Erotic Explicit Cues	4.06 (0.80)	3.82 (0.84)	3.76 (1.05)	9.11 ***	0.13
Sensory Explicit Cues	2.94 (0.91)	2.87 (0.98)	3.08 (0.99)	1.33	0.04

Note: *M* = Mean; *SD* = Standard Deviation, *F* = Analysis of variance, *p* = significance value, *η^2^* = eta squared, *** *p* < 0.001, BSAS—Brief Sexual Attitudes Scale; CSDS—Cues for Sexual Desire Scale.

**Table 3:** Comparison of Brief Sexual Attitudes Scale and Cues of Sexual Desire Scale means and subscales concerning sexual orientation.

Instruments	Heterosexual	Other	*t* (804)	Cohen’s *d*
	*M (SD)*	*M (SD)*		
**BSAS**				
BSAS total	3.25 (0.53)	3.50 (0.47)	−4.659 ***	−0.494
Permissiveness	2.76 (0.91)	3.32 (0.80)	−5.868 ***	−0.622
Sexual practices	4.52 (0.68)	4.56 (0.67)	−0.515	−0.055
Instrumentality	2.68 (0.79)	2.84 (0.723)	−1.924	−0.204
Communion	3.92 (0.67)	3.87 (0.78)	0.731	0.078
**CSDS**				
CSDS total	3.46 (0.51)	3.62 (0.47)	−2.940 **	−0.312
Visual Proximity Cues	2.34 (0.89)	2.62 (0.90)	−3.038 **	−0.322
Emotional Bonding Cues	4.40 (0.63)	4.34 (0.63)	0.786	0.083
Romantic Implicit Cues	3.45 (0.81)	3.63 (0.76)	−2.134 *	−0.226
Erotic Explicit Cues	3.98 (0.84)	4.20 (0.72)	−2.583 **	−0.274
Sensory Explicit Cues	2.90 (0.93)	3.16 (0.87)	−2.637 **	−0.279

Note: *M* = Mean; *SD* = Standard Deviation, *t* = Student’s *t*-test, *d* = Cohen´s *d* effect size, * *p* < 0.05, ** *p* < 0.01, *** *p* < 0.001, BSAS—Brief Sexual Attitudes Scale; CSDS—Cues for Sexual Desire Scale.

**Table 4:** Comparison of Brief Sexual Attitudes Scale and Cues for Sexual Desire Scale means and subscales concerning relationship duration.

Instruments	No Relation	<6 Months	6–12 Months	12–24 Months	>24 Months	*F* (804)	*η^2^*
	*M* (*SD*)	*M* (*SD*)	*M* (*SD*)	*M* (*SD*)	*M* (*SD*)		
**BSAS**							
BSAS total	3.38(0.52)	3.39(0.51)	3.20(0.52)	3.26(0.53)	3.19(0.52)	6.41 ***	0.17
Permissiveness	3.14(0.84)	3.07(0.90)	2.75(0.85)	2.69(0.91)	2.57(0.91)	54.43 ***	0.28
Sexual practices	4.45(0.76)	4.55(0.73)	4.42(0.70)	4.55(0.64)	4.60(0.58)	4.32	0.11
Instrumentality	2.76(0.75)	2.62(0.85)	2.55(0.80)	2.82(0.91)	2.65(0.76)	4.40	0.09
Communion	3.79(0.70)	4.02(0.61)	3.93(0.87)	3.94(0.65)	4.00(0.66)	7.05 **	0.14
**CSDS**							
CSDS total	3.41 (0.54)	3.62 (0.45)	3.60 (0.49)	3.56 (0.51)	3.48 (0.48)	3.56 **	0.13
Visual Proximity Cues	2.56 (0.83)	2.58 (0.91)	2.15 (0.73)	2.41 (0.98)	2.19 (0.89)	25.03 ***	0.20
Emotional Bonding Cues	4.16 (0.68)	4.35 (0.63)	4.51 (0.57)	4.61 (0.50)	4.51 (0.56)	24.81 ***	0.28
Romantic Implicit Cues	3.34 (0.83)	3.65 (0.74)	3.68 (0.73)	3.58 (0.84)	3.49 (0.78)	10.10 **	0.14
Erotic Explicit Cues	3.90 (0.86)	4.21 (0.63)	4.33 (0.81)	4.05 (0.87)	3.99 (0.81)	11.62 **	0.15
Sensory Explicit Cues	2.95 (0.93)	3.17 (0.81)	3.04 (0.90)	2.80 (0.92)	2.90 (0.95)	5.84	0.09

Note: *M* = Mean; *SD* = Standard Deviation, *F* = Analysis of variance, *η^2^* = eta squared, ** *p* < 0.01, *** *p* < 0.001, BSAS—Brief Sexual Attitudes Scale; CSDS—Cues for Sexual Desire Scale.

**Table 5:** Comparison of Brief Sexual Attitudes Scale and Cues of Sexual Desire Scale means and subscales concerning the nature of the relationship.

Instruments	No Relation	No Commitment	Commitment	*F* (804)	*η^2^*
	*M* (*SD*)	*M* (*SD*)	*M* (*SD*)		
**BSAS**					
BSAS total	3.38 (0.52)	3.32 (0.50)	3.22 (0.53)	4.45 ***	0.14
Permissiveness	3.13 (0.84)	2.79 (0.82)	2.65 (0.92)	41.02 ***	0.25
Sexual practices	4.44 (0.76)	4.59 (0.69)	4.57 (0.62)	2.94 *	0.09
Instrumentality	2.76 (0.76)	2.76 (0.84)	2.66 (0.80)	1.94	0.06
Communion	3.79 (0.70)	4.05 (0.56)	3.98 (0.68)	7.64 ***	0.14
**CSDS**					
CSDS total	3.41 (0.54)	3.52 (0.50)	3.52 (0.49)	2.23	0.10
Visual Proximity Cues	2.54 (0.83)	2.35 (0.84)	2.27 (0.92)	13.03	0.14
Emotional Bonding Cues	4.16 (0.68)	4.29 (0.65)	4.53 (0.55)	24.31	0.28
Romantic Implicit Cues	3.34 (0.83)	3.67 (0.77)	3.54 (0.79)	8.25	0.13
Erotic Explicit Cues	3.89 (0.86)	4.15 (0.80)	4.06 (0.80)	5.70	0.10
Sensory Explicit Cues	2.94 (0.92)	2.95 (1.03)	2.92 (0.92)	0.07	0.01

Note: *M* = Mean; *SD* = Standard Deviation, *F* = Analysis of variance, *η^2^* = eta squared, * *p* < 0.05,*** *p* < 0.001, BSAS—Brief Sexual Attitudes Scale; CSDS—Cues for Sexual Desire Scale.

**Table 6:** Comparison of Brief Sexual Attitudes Scale and Cues of Sexual Desire Scale means and subscales concerning current sexual practices and partners.

Instruments	No Sex	No Sexual Partner	One Sexual Partner	>One Sexual Partner	*F* (804)	*η^2^*
	*M (SD)*	*M (SD)*	*M (SD)*	*M (SD)*		
**BSAS**						
BSAS total	3.14 (0.53)	3.57 (0.48)	3.24 (0.52)	3.72 (0.40)	14.61 ***	0.26
Permissiveness	2.70 (0.91)	3.45 (0.74)	2.72 (0.91)	3.33 (0.57)	57.16 ***	0.29
Sexual practices	4.44 (0.72)	4.56 (0.72)	4.54 (0.66)	4.63 (0.51)	1.54	0.07
Instrumentality	2.60 (0.74)	2.91 (0.76)	2.67 (0.80)	3.05 (0.74)	9.40 **	0.14
Communion	3.71 (0.75)	3.83 (0.66)	3.99 (0.77)	4.09 (0.63)	10.11 ***	0.16
**CSDS**						
CSDS total	3.30 (0.59)	3.53 (0.49)	3.51 (0.48)	3.60 (0.61)	5.67 ***	0.17
Visual Proximity Cues	2.43 (0.85)	2.61 (0.88)	2.29 (0.89)	2.87 (0.86)	14.42 ***	0.15
Emotional Bonding Cues	4.19 (0.70)	4.23 (0.72)	4.48 (0.59)	4.13 (0.83)	13.74 ***	0.21
Romantic Implicit Cues	3.26 (0.94)	3.41 (0.81)	3.54 (0.76)	3.71 (0.71)	9.17 **	0.13
Erotic Explicit Cues	3.57 (0.99)	4.14 (0.61)	4.08 (0.78)	4.11 (0.87)	32.15 ***	0.24
Sensory Explicit Cues	2.71 (0.92)	3.23 (0.83)	2.92 (0.92)	3.19 (1.22)	18.28 ***	0.16

Note: *M* = Mean; *SD* = Standard Deviation, *F* = Analysis of variance, *η^2^* = eta squared, ** p < 0.01 *** p < 0.001, BSAS—Brief Sexual Attitudes Scale; CSDS—Cues for Sexual Desire Scale.

**Table 7:** Correlations between Cues for Sexual Desire Scale and Brief Sexual Attitudes Scale.

Instruments	BSAS Total	Permissiveness	Sexual Practices	Instrumentality	Communion
**CSDS total**	0.324 **	0.208 **	0.049	0.246 **	0.287 **
Visual Proximity Cues	0.330 **	0.308 **	−0.078 *	0.292 **	0.089 *
Emotional Bonding Cues	−0.062	−0.174 **	0.110 **	−0.019	0.166 **
Romantic Implicit Cues	0.144 **	0.017	0.035	0.174 **	0.226 **
Erotic Explicit Cues	0.347 **	0.298 **	0.075 *	0.134 **	0.259 **
Sensory Explicit Cues	0.369 **	0.336 **	0.047	0.179 **	0.211 **

* p < 0.05, ** p < 0.01, BSAS—Brief Sexual Attitudes Scale; CSDS—Cues for Sexual Desire Scale.

**Table 8:** Multiple linear regression for the total and Permissiveness Brief Sexual Attitudes Scale.

Variables	*R*²	*B*	*β*	*t*
**Total BSAS ^1^**				
Age	0.252	−0.004	−0.072	−2.225 **
Sexual orientation		0.088	0.108	3.422 ***
Relation nature		−0.104	−0.185	−4.525 ***
Sexual practices		0.083	0.125	3.065 **
Visual Proximity Cues (CSDS)		0.115	0.194	5.890 ***
Erotic Explicit Cues (CSDS)		0.093	0.145	3.689 ***
Sensory Explicit Cues (CSDS)		0.120	0.210	5.460 ***
**Permissiveness BSAS ^2^**				
Age	0.310	−0.013	−0.149	−4.742 ***
Sexual orientation		0.209	0.148	4.882 ***
Relation nature		−0.208	−0.214	−5.295 ***
Sexual practices		0.132	0.114	2.905 ***
Sexual practices		0.198	0.192	5.409 **
Visual Proximity Cues (CSDS)		−0.181	−0.125	−3.679 ***
Emotional Bonding Cues (CSDS)		−0.145	−0.128	−3.504 ***
Romantic Implicit Cues (CSDS)		0.188	0.169	4.303 ***
Erotic Explicit Cues (CSDS)		0.189	0.192	5.174 ***
Sensory Explicit Cues (CSDS)		−0.013	−0.149	−4.742 ***

Note: *R*²: determination coefficient, *β*: standardized beta coefficient, *t*: Student’s *t*-test, ** *p* < 0.01, *** *p* < 0.001, BSAS—Brief Sexual Attitudes Scale; CSDS—Cues for Sexual Desire Scale. ^1^ Total BSAS: *F*(7, 787) = 37.851, *p* < 0.001, Adjusted *R*² = 0.286. ^2^ Permissiveness BSAS: *F*(9, 787) = 39.215, *p* < 0.001, Adjusted *R*² = 0.302.

## Data Availability

As part of consenting to the study, survey respondents were assured that raw data would remain confidential and no personal data would be shared. The database will be made available upon request to angelamtleite@gmail.com.
